# Prevalence and Consequences of Aggression and Violence towards Nursing and Care Staff in Germany—A Survey

**DOI:** 10.3390/ijerph15061274

**Published:** 2018-06-15

**Authors:** Anja Schablon, Dana Wendeler, Agnessa Kozak, Albert Nienhaus, Susanne Steinke

**Affiliations:** 1 Competence Centre for Epidemiology and Health Services Research for Healthcare Professionals (CVcare), University Medical Centre Hamburg-Eppendorf (UKE), 20246 Hamburg, Germany; a.kozak@uke.de (A.K.); Albert.Nienhaus@bgw-online.de (A.N.); su.steinke@uke.de (S.S.); 2 Department for Occupational Medicine, Hazardous Substances and Public Health Accident Insurance and Prevention in the Health and Welfare Services (BGW), 22089 Hamburg, Germany; dana.wendeler@bgw-online.de

**Keywords:** violence, aggression, nurses, care staff, stress perception, professional management of violent attacks

## Abstract

Acts of aggression by patients or clients are a part of the average working day for many Health care employees. The objective of the survey was to study the frequency and nature of violence and the handling of aggressive behavior by facility management. The cross-sectional study was conducted in 2017, 81 different healthcare facilities and 1984 employees participated. The questionnaire encompassed socio-demographic details, the frequency of physical violence and verbal abuse, consequences of violence and the stress of employees. In the previous twelve months, 94.1% of the employees in the survey had experienced verbal abuse and 69.8% had experienced physical aggression. Acts of aggression were most commonly encountered in hospitals and residential facilities for the disabled. One third of the employees felt under high levels of stress as a result of the incidents. If the workplace prepares effectively, however, this reduces the perceived stress odds ratio (OR) 0.6, 95% CI 0.4–0.8). Violence and aggression are very common. Healthcare facilities are increasingly dealing with this topic. Awareness raising is likely to lead to higher incident reporting rates. Good preparation and an open approach to the topic in the facilities have a positive effect on the feeling of stress and work ability.

## 1. Introduction

Aggressive acts towards employees in the healthcare and welfare sector by patients, clients, residents, and relatives is a very sensitive topic that is frequently stigmatized. The International Labor Organization [1] defines violence and aggression at work on the basis of criteria developed by the European Union [2,3]. These state that violence comprises any action, incident or behavior where staff is abused, threatened, assaulted or harmed in circumstances related to their work, including commuting to and from work, and involving an explicit or implicit challenge to their safety, well-being or health”.

Compared with other groups of employees, people in healthcare are at a higher risk of being confronted with violence by patients/clients or relatives in the workplace. An analysis of accidents in the workplace requiring reporting in 2016 showed that the proportion assigned to violence by other people was 1.4%. Employees with patient/customer contact are at particularly high risk. A good 31% (*n* = 3252) of all acts occurred among employees in hospitals and care homes [4]. Other studies found high prevalence rates among employees in psychiatric institutions and in A&E departments [5,6]. The consequences of violence vary widely. Lanctot and Guay [7] identified in their systematic literature review seven categories of consequences of workplace violence such as physical, psychological, emotional, work functioning, relationship with patients/quality of care, social/general, and financial.

In addition to potential physical injuries, the focus in particular is on psychological consequences. These may restrict the satisfaction, health, and performance of employees over the longer term [8,9] and result in lower job satisfaction and an increase in the number of sick days [10].

Those HCW affected often believe that violence by patients/clients or relatives is a normal part of the job and therefore do not report it to their employer or file accident reports. The study conducted by Schablon et al. in 2009 discovered that 56% of the nursing and care employees in the survey had experienced physical violence and 78% had experienced verbal abuse. Around one third of those affected felt under high levels of stress as a result. Employees in inpatient geriatric care and facilities for people with disabilities were the most likely to be affected by violence and aggressive acts [11]. Demographic change continuously leads to an increase in the number of patients with dementia in nursing homes for the elderly [12]. Dementia care can be very challenging due to the cognitive and functional decline of patients; however, it may be suggested that nurses’ low level of knowledge of patients’ needs and an unfavorable attitude towards dementia care can have a negative impact on care, patient safety, and symptom management [13].

With regard to the frequency and impact of violence on employees, violence prevention through information and professional dealing is continuing to gain significance. The aim of this study was to determine the current statistics on the types and consequences of aggressive acts on employees in hospitals, inpatient and outpatient geriatric care homes and in facilities for people with disabilities in Germany. In addition, it also investigated how attacks and aggressive behavior encountered by nursing and care staff were dealt with.

## 2. Materials and Methods

**Figure 1:**
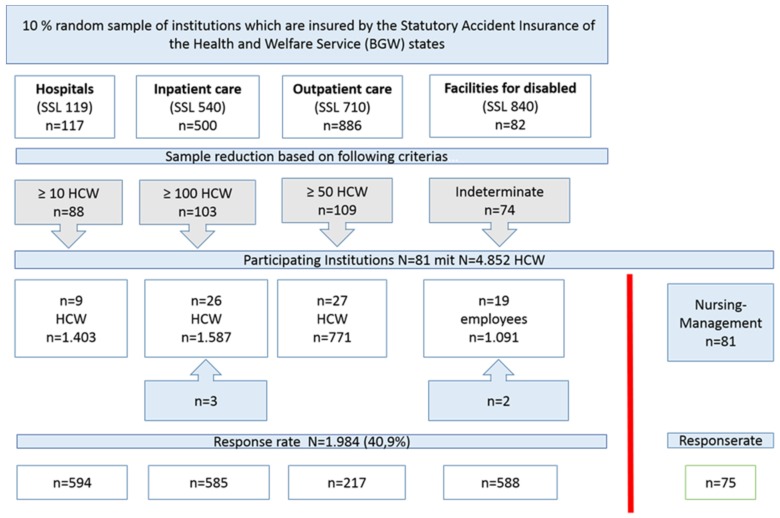
Flow chart study population.

This cross-sectional study was conducted in 2017. A 10% random sample was taken of workplaces in the four categories of hospitals, inpatient and outpatient geriatric care and facilities for people with disabilities, which are insured by the Statutory Accident Insurance of the Health and Welfare Service (BGW) and come from four different German states (Bavaria, Berlin, Mecklenburg-Western Pomerania, and North Rhine-Westphalia). This resulted in a total of 1585 facilities. To reduce the sample, a minimum number of employees was defined for the respective industry, (Figure 1) leaving 374 institutions that were then contacted and invited to participate.

A total of 81 workplaces participated in the study with a potential of around 5000 employees who met the required patient/client/resident contact for a study participation. Each employee received a questionnaire, which was returned directly to the evaluating institution in a self-addressed envelope.

Another additional data basis was the survey of the respective management staff of each institution, which was conducted and evaluated separately from the survey of employees. The data was collected in writing via structured questionnaires. The data collection questionnaire for employees included questions about a range of topics.

The first block of topics included the socio-demographic data about the person, including their age, sex, and the country in which their parents were born. With regard to their professions, the participants were asked about the sector affiliation of their institution and the area of healthcare in which they work. We also asked about professional qualifications, the number of years working in nursing/care, the number of working hours per week and the frequency of early, late, and night shifts per month.

The second block of questions dealt with incidences of verbal/physical violence and aggression by patients/clients/residents in the previous twelve months. The experience of violence and aggressive behavior and the measures taken for managing such situations were recorded via a self-administered questionnaire which was drawn up along Staff Observation Aggression Scale-Revised (SOAS-R) guidelines by Nijman et al. [14]. We recorded the frequency, type, and target of the attack, as well as the measures taken to stop the aggression. There were then some questions on mental stress resulting from violence and aggression. The participants evaluated the level of mental stress they experienced as a result of the attack, the emotions they associated with it, and the extent to which it had affected their work with their patients/clients/residents. In the case of physical attacks, we asked about possible impairments. In addition, we collected information as to whether there had been instances of psychological aggression by colleagues or verbal and physical attacks by relatives of patients/clients/residents in the previous twelve months.

The next block of questions dealt with how violence is handled as a topic within the institutions. We collected data on whether the participants of the survey felt that they had received support after experiencing violence and aggression, and if so, from whom. The participants were to evaluate the level of support they received from colleagues and superiors in managing the aggressive acts and whether they felt well prepared for situations involving aggressive or violent patients/clients/residents by their institution. Data was also collected on whether the institution offered methods of dealing with violent and aggressive acts, whether de-escalation training was carried out and whether the participant was aware that violent attacks were classed as occupational accidents and that preparatory sessions with a trauma therapist could be claimed from the accident insurance institution. Questions about reporting behavior collected data on whether the survey participants reported the attacks and, if they did, to whom, as well as whether the attacks were systematically documented within the institution. A comment box allowed participants to express recommendations, requests, and comments about improving the work environment with regard to violent and aggressive acts experienced in the workplace.

Finally, there were some questions on working capacity. Data was collected on the person’s assessment of current working capacity with regard to the psychological demands of their work. We also asked about the degree to which the employees believed they would be able to continue doing their current job in the next two years, based on their current health status. The questions were taken from the Work Ability Index (WAI) [15]. We also asked whether the participant was considering leaving their profession (Copenhagen Psycho-Social Questionnaire (COPSOQ)) [16].

The questionnaire used in this study was tested for comprehensibility and suitability in preliminary trials [11,17].

### 2.1. Questionnaire for Management

The additional questionnaire for management staff included questions about the institution, such as sector affiliation, area of healthcare, number of patients/clients/residents at the time of the survey, and current number of nursing and care staff. This part of the survey was evaluated separately from the employees’ questionnaire. Furthermore, the participants were also asked which shifts were most likely to experience violent and aggressive acts. Finally, there were some questions on prevention programs and follow-up care at the institution, as well as the frequency of their use by the staff affected. We asked the participants to assess whether or not they thought the measures in place at the institution were effective. Questions about reporting behaviors recorded whether violent attacks in the institution were documented systematically and reported to superiors or accident insurers. Data was collected on whether the institution had carried out a risk assessment with regard to violent and aggressive acts.

### 2.2. Ethics

The Hamburg Medical Chamber ethics committee approved the study (reference number: PV5405). Participation was voluntary. To ensure anonymity, no names or other identifiers were used.

### 2.3. Statistical Methods

The data was evaluated using SPSS version 23 ((IBM Corp. Released 2015. IBM SPSS Statistics for Windows, Version 23.0. Armonk, NY: IBM Corp.). The samples were presented using descriptive statistics, while distributions of the variables by category were analyzed using the chi-squared test. For the logistic regression models, the original visual, ten-stage analogue scales for estimating stress level, social support and in-house training were summarized as three-stage variables with the criteria insufficient or low (1–3), average (4–7), and good or high (8–10). To determine influencing factors in the prevalence of violence, stress perception after an attack and gauging work capacity, we calculated the odds ratio (OR) and 95% confidence intervals (CIs) using logistic regression models. The modelling was done backwards stepwise using the Hosmer–Lemeshore test. Variables with *p*-values higher than >0.1 were successively excluded.

## 3. Results

A total of 1984 out of 4852 employees from hospitals, inpatient and outpatient geriatric care and facilities for people with disabilities took part in the study. This corresponds to a response rate of 40.9%. Categorized by sector, 217 employees took part from outpatient geriatric care, 585 from inpatient geriatric care, 594 from hospitals, 234 from occupational therapy institutions, and 310 from residential facilities for people with disabilities. The share of women was 79%. Among the respondents, 34% were employed as nursing or care staff without a managerial role. 20.8% were employed in peripheral healthcare roles (e.g., as nursing assistants, care assistants or staff without nursing qualifications). The category of social professions (18.5%) includes educationalists, social workers, educators, and remedial teachers (Table 1).

The prevalence of verbal abuse and physical violence towards nurses in hospitals decreased continuously from the age of 30 years, just as the prevalence of physical violence in nursing care for the elderly and in facilities for the disabled. In the <20-year-old age group, nurses in elderly care are notably often affected by verbal abuse and physical violence. In contrast to all other care sectors, female HCW in geriatric care are more frequently affected by verbal abuse and physical violence than men (no table).

### 3.1. Frequency of Physical and Verbal Aggression

**Figure 2:**
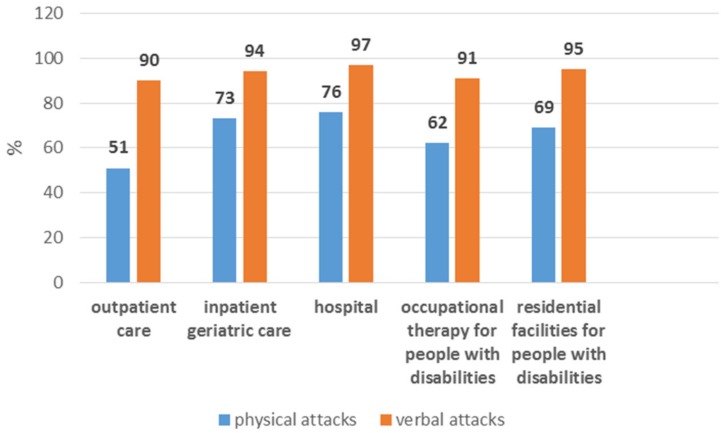
Frequency of physical and verbal attacks differ by workplace among employees who have experienced violence in the last 12 months.

Of the 1984 study participants, 20.5% said they have not experienced violence in the last 12 months. 94.1% of the respondents who reported violence in the past twelve months said that they had suffered verbal abuse and 69.8% had experienced physical violence. Employees in inpatient geriatric care were the most likely to be affected by daily physical and verbal attacks. The perpetrators were mainly patients or clients (verbal attacks 72%, physical attacks 96%). Verbal attacks were most frequently reported by hospital employees, followed by employees in residential facilities for people with disabilities. The highest levels of physical violence were experienced by those working in a hospital setting (76%), followed by inpatient geriatric care (Figure 2).

Hospitals are the most heterogeneous group. In some of the facilities, the survey was performed on individual wards only and did not cover the entire house. The departments included in the survey varied in the different hospitals. Table 2 gives an overview of the frequency of physical and verbal attacks and the response rates for each hospital.

Both physical (58% vs. 42%) and verbal (61% vs. 39%) attacks were more common in general wards than in psychiatry/geriatric psychiatry wards. 61% of employees working in psychiatry had taken part in de-escalation training, but only 39% of employees on general wards had done so. Daily physical attacks were experienced by 10% of all participants, and verbal attacks and aggression by 21%. There were no differences between people with or without migrant backgrounds (no table).

### 3.2. Violent Attacks and Their Consequences

The violent and aggressive acts primarily comprised insults, pinching and scratching, hitting, and threats. Objects were used most often in hospitals and in residential facilities for people with disabilities (36.8% and 35.6%). Sexual harassment was reported by all areas, with geriatric care showing the highest incidence at 18.1% (Table 3). In most cases, the target of the aggression was the respondent him/herself (69%) or other patients/clients/residents (54%). Furthermore, 25% of employees reported having witnessed self-harm.

Incidents primarily occurred during the day shift (47.6%). Most respondents reacted with annoyance, anger, fear, helplessness, or disappointment. As a result, those affected become more careful, vigilant and tense, take less enjoyment in their work and in interactions with patients/clients/residents. With regard to physical impairments, 42% of those affected stated that they experienced pain for more than ten minutes, 58% had visible injuries, and 19% had to seek medical attention. Table 4 presents the consequences of violence by workplace. After attacks, annoyance and anger were most commonly experienced by employees in hospitals and facilities for people with disabilities. These areas of work were also most likely to experience pain lasting longer than ten minutes and requiring medical attention (Table 4).

With regard to the question as to which measures had been adopted to resolve the situation, employees most commonly cited a conversation with the patient/client/resident (81.9%), followed by gently walking away from the aggressor (51.6%), requesting the aggressor to change his/her behavior (46.6%), removing the aggressor (44.5%) and requesting assistance from other members of staff (39.1%). 32.5% of the respondents stated that the aggressor was calmed using appropriate medication. 11.5% called the police. Two thirds (63.8%) did not feel well prepared by the institution for such attacks (Table 1). Social support was received primarily from colleagues (81.2%) (no table).

### 3.3. How Institutions Deal with the Topic

The question as to whether such cases are systematically documented by the institution received a “yes” response from 55% of the participants. 85% of employees reported the attack: in 94.6% of cases to a superior and in 9% to the accident insurer. The fact that such attacks are classed as accidents in the workplace and that there is therefore eligibility for preparatory sessions paid for by the accident insurer was unknown to 60.2% of the employees (no table). 41.8% of respondents had taken part in de-escalation training (Table 1), with respondents working at hospitals and in residential care being more likely to have done so (51.2% and 52.8%), with only 25% and 34.1%, respectively, having done so in outpatient and inpatient geriatric care. The institutions primarily offer case assessments and supervision (58.5%). Guidelines on how to act when confronted with aggressive patients/clients/residents were available according to 27.3% of the employees, with follow-up care discussions (26.4%) and technical emergency systems (15.1%) being mentioned less often. 22% of those in the survey stated that there were no such services offered by their institution.

### 3.4. Attacks by Relatives and Colleagues

Physical attacks by relatives had been experienced by 3.7% of respondents and verbal attacks by relatives by 32.1%.

Another type of violence and aggression is horizontal violence from colleagues. 41% of the HCW stated that they had experienced psychological aggression, such as defamation or negative gossip from colleagues.

### 3.5. Risk Factors for Physical and Verbal Attacks

Women are less affected by verbal abuse than men (OR 0.5, 95% CI 0.2–0.9). The risk of receiving verbal attacks is highest in the 40–49 age group (OR 1.4, 95% CI 0.7–3.0). This result is not statistically significant, however. The risk of experiencing physical violence decreases with age (>60 years OR 0.6, 95% CI 0.3–0.9). Compared to outpatient care, working in hospitals, inpatient geriatric care or in occupational therapy/residential facilities for people with disabilities is associated with a greater risk of experiencing physical violence. All professional groups showed an increased odds ratio for experiencing verbal attacks in comparison to peripheral medical positions. However, the difference is only statistically significant between nursing and care staff with managerial roles (OR 2.3, 95% CI 1.2–4.4) (Table 5).

### 3.6. Risk Factors for High Stress Perception

Of the 1522 employees who had experienced verbal and/or physical violence and aggression over the previous twelve months, one third said this resulted in high levels of stress. The highest stress perception came from staff at hospitals (44%) and residential facilities for people with disabilities (40%), followed by staff at occupational therapy facilities (33%), with 27% of those working in inpatient and outpatient geriatric care feeling under high levels of stress as a result. Employees at residential facilities for people with disabilities (OR 1.7, 95% CI 1.0–2.8) and in hospitals (OR 2.1, 95% CI 1.3–34) felt under more stress than those in outpatient care.

**Figure 3:**
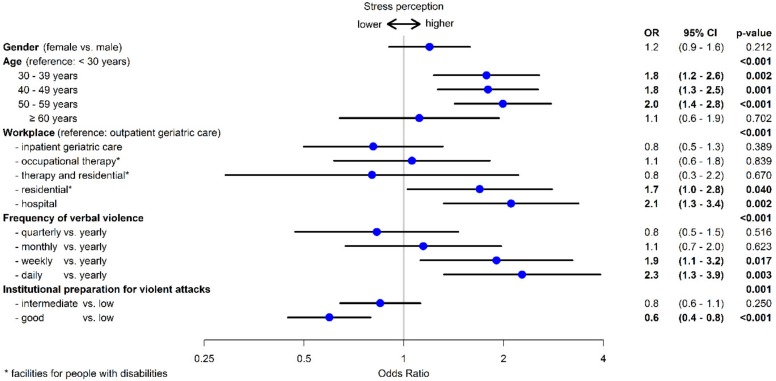
Risk factors for high stress perception (*n* = 1522).

Women feel under higher stress, although this result is not statistically significant. Compared to younger employees, the risk for high stress perception increases with age (Figure 2). For employees aged over 60, the stress perception decreases again. The more frequently employees are exposed to verbal abuse, the more stressed they feel. Where verbal abuse is experienced on a daily basis, the OR rises to 2.3 (95% CI 1.3–39). The experience of physical violence and the participation in de-escalation training had no influence on stress perception. Where the institution provided good preparation for such events, the employees’ stress perception decreased (OR 0.6, 95% CI 0.4–0.8) (Figure 3).

### 3.7. Subjective Evaluation of Work Ability

**Figure 4:**
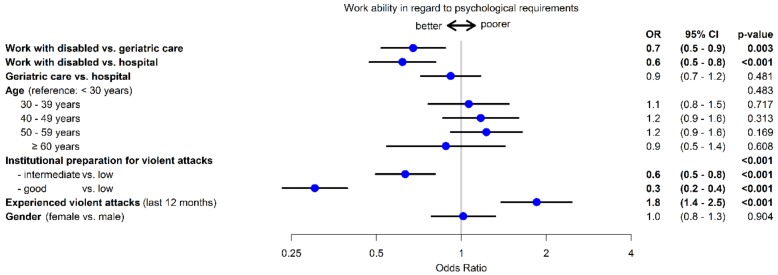
Risk factors for work ability with regard to the psychological demands of the job.

With regard to the psychological requirements of their job, 70.2% of participants evaluated their work capacity as good to very good. Furthermore, 79.5% believed that they would still be able to do their job in two years’ time. 89.3% had only occasionally or never thought about giving up their job. Risk factors for decreased work capacity are experiencing violent attacks (OR 1.8, 95% CI 1.4–2.5) and increasing age. However, preparation for possible violent attacks appears to have a protective effect (OR 0.3, 95% CI 0.2–0.4). Working with people with disabilities also has a protective effect on work capacity as compared with working in geriatric care or in hospitals (Figure 4).

### 3.8. Results for Management 

Of the 81 participating institutions, 75 nursing management staff filled in the questionnaire. In response to the question as to which preventive programs are offered by the institutions, they listed most frequently case assessments/supervision (90.9%), follow-up care discussions (68.9%), guidelines for recommended action (47.3%), de-escalation training (37.8%) and technical emergency systems (27.0%). Only 47.9% stated carrying out risk assessments on the topic of violence. According to the information provided by the managerial staff, most occurrences took place during the day shift (56.8%). Over 62% of institutions systematically recorded such events. 66.7% of managerial staff stated that they reported incidents. 56.6% were reported to accident insurers, with reports to the body responsible for the institution occurring more frequently (73.6%). 60.8% of managerial staff are aware that violent attacks are classed as occupational accidents and that preparatory sessions can be claimed from the accident insurer. In response to the question as to which services are used by employees affected, they stated support from colleagues (frequent, 87.7%), support from the supervisor (frequent, 78.4%), case assessments (frequent, 77.0%), and supervision (frequent, 31.4%). According to the managerial staff, only 17.8% of employees frequently took part in de-escalation training after an event (no table).

## 4. Discussion

This is the second time since 2009, that a study is being conducted in Germany investigating the frequency of verbal and physical violence and aggressive acts perpetrated against employees in the healthcare and welfare sector. It revealed that violence and aggressive acts are very common and have increased by 10% to 20% as compared with 2009 [11]. In our study, 94.1% of the employees stated that they had experienced verbal abuse and 69.8% had experienced physical violence in the past twelve months. Furthermore, 40.8% of the respondents stated, that they had experienced psychological aggression from colleagues. Violent attacks by relatives, however, were rare. Having said this, there are variations between the different workplaces.

The study by Groenewold et al. [18] from the US also determined an increase in violent attacks between 2012 and 2015: the incidence rate in participating hospitals increased from 4.4/1000 full-time employees to 7.7/1000 between 2012 and 2014, while the rate was 7.2% in 2015. In our study, the employees at hospitals (97%) and residential facilities for people with disabilities (95%) were most affected by verbal abuse, followed by employees working in inpatient geriatric care (94%). Physical violence was most commonly experienced by staff at hospitals and in inpatient geriatric care.

Furthermore, employees in inpatient geriatric care were the most likely to be affected by daily physical and verbal attacks. As a result of the demographic change, the number of patients with dementia continues to increase, especially in nursing homes for the elderly. Almost 1.6 million people with dementia live in Germany; two thirds of them are affected by Alzheimer’s disease [12]. These patients in particular represent a risk group for aggression, as well-intentioned acts of care can be misinterpreted and therefore lead to aggressive reactions.

Targeted prevention programs for inpatient geriatric care facilities are therefore also necessary. Outpatient care showed the lowest rates of physical violence. In comparison to results from 2009, there was a significant increase in verbal and physical attacks in hospitals. In the study from 2009 [11], the prevalence rate for physical violence in hospitals was 56% vs. 73% in 2017, with this increasing from 79% to 97% for verbal abuse. The awareness for the topic in the last years in Germany has likely resulted in a change in perception of such attacks. What was once considered commonplace in these professions is now seen as something that does not have to be tolerated. We assume, that this resulted in higher reporting figures. More than 40% of the participants took part in de-escalation trainings that were in particular offered by the employers’ liability insurance association as part of their many preventive measures in the last years.

In both German cross-sectional studies from 2009 and 2017, outpatient care was the least affected by violent incidents. This has also been confirmed by a study from Poland. This showed that nurses in inpatient care were more frequently affected by violence than employees in the outpatient sector [19]. Other studies investigating the frequency of violent attacks determined, that the highest prevalence rates were in psychiatric institutions and A&E departments. A study from Italy looked at the frequency and characteristics of violence in various sectors and professional groups [20]. Here, most cases were shown to occur in psychiatry (86%), followed by A&E departments (71%), and geriatric care (57%). Non-physical violence was significantly more frequent on geriatric wards and in A&E departments [20]. Hospitals are a very heterogeneous group within the variety of working sectors that were included in this study. However, the prevalence of violence in both psychiatric and general wards is high. However, employees in the psychiatric sector are better prepared for assaults than employees in other areas where no increased risk is assumed. This clearly shows that violence prevention is also very important in areas with unexpected risks for violence; i.e., in facilities where more patients with dementia are being treated.

In their meta-analysis, Edward et al. [21] investigated factors playing a role in violent attacks. This analysis showed that employees in psychiatry were significantly more frequently the subjects of physical violence than employees on other wards (OR 2.91, 95% CI 2.20–3.85). The study by Hylen et al. [22] from 2017 also clearly showed that employees on psychiatric wards were frequently exposed to violence (87%).

Studies looking at the frequency of violent attacks in disabled care also revealed high prevalence rates [11,17]. In the 2012 study by Schablon et al. [11], employees at residential facilities for people with disabilities were most likely to be affected by verbal abuse (86%). Franz et al. [17] found a prevalence rate of 77.4% for physical violence and 42% for verbal abuse in occupational care facilities for people with disabilities. The high prevalence rates in this workplace are confirmed in this study.

### 4.1. Violent Incidents by Individual Profession

In addition to nursing and care staff, participants in our study included other professional groups, such as social workers/educationalists, educators, and people in peripheral healthcare professions, who have regular contact with patients, clients and residents. As described in other studies, nursing and care staff were the most frequently affected by violent incidents. However, violent and aggressive acts are also commonplace in social professions. In the Italian study, nurses were most commonly affected by violence (67%), followed by carers (18%) [20].

Magnavita and Heponiemi [23] also found that nurses are more likely to encounter aggressive behavior due to the increased amount of time they spend caring for patients.

Other studies showed similar results. A study from the USA showed that carers (OR 14.89, 95% CI 10.12–21.91) and nurses (OR 8.05, 95% CI 6.14–10.55) experienced the highest prevalence rates of injuries resulting from violence [18]. In a study by Cheung and Yip [24] nurses were shown to have a significantly higher risk of physical and verbal assault as compared with doctors. Our study showed that nursing and working as a carer with a managerial role is a risk factor for violent attacks. This might come along with the fact, that in Germany carers with a managerial role are also engaged in patient care. Due to their professional experience they often take over the care of difficult patients and are summoned for help, once situations escalate.

The results underline the importance of preventive measures in clinics and geriatric care institutions, but also that violent episodes play a major role in social professions, such as in disabled care, and that prevention is also an important topic in these areas, too.

### 4.2. Horizontal Violence

Violence among colleagues (horizontal) is also common in the nursing and care professions. Horizontal violence includes bullying, screaming, constant criticism, eye-rolling, denigrating talk, sabotage and ignoring someone [8,25]. Horizontal violence can lead to stress, depression, anxiety, job dissatisfaction and sleep disorders, but may also have a negative impact on patient care and the entire organization as a whole [26,27]. In our study, just under 41% of the respondents stated that they had experienced psychological violence from colleagues. In order to break the cycle of psychological stress and horizontal violence, interventions are also necessary among colleagues in the workplace to reduce psychosocial stress and aggressive behavior. Furthermore, violence among colleagues threatens the well-being of HCW and should be regularly tracked with other forms of violence [23,28].

### 4.3. Age and Gender

Some studies showed that women were more often affected by violence than their male colleagues [18,21,29]. Having said this, men were more often the targets of physical violence because they are more likely to be stationed in high-risk environments [21]. The study by Magnavita and Heponiemi [23] showed that physical aggression and also threats directed towards males were slightly more frequent than towards female HCW, whilst females were more often victims of harassment.

The study by Zampieron et al. [29] showed that 49% of nursing and care staff were affected by violent episodes, predominantly women (52%). The study from the US also showed, that women experienced violent attacks more often than their male colleagues (66.4% vs. 25.9%). In the German study from 2009, however, no heightened risk was determined for women, and this is also borne out by the current study [11]. A Polish study also found no significant differences by gender [19]. Some studies showed that younger employees had a higher risk of physical attacks than their older colleagues [6,11,18]. Our study also showed that the risk of violent attack decreases with age.

### 4.4. Consequences of Violent Experiences and Stress Perception

The consequences of violent attacks can be manifold. The focus is often on the psychological consequences. Post-traumatic stress disorder (PTSD), time off work, decreasing motivation, lower job satisfaction, loss of confidence, questioning one’s own identity and anxiety are potential reactions that can restrict the happiness and performance of the person affected over the long term [8,10,30]. One study looking at psychiatric nursing staff investigated the correlation between violent attacks and stress: 88.1% of employees had been affected by verbal abuse and 58.4% by physical violence. The violent attacks correlated with job stress, general satisfaction, and PTSD [31].

In our study, those affected reacted with annoyance, anger, anxiety, helplessness, and disappointment. As a result, they have become more careful, vigilant, and tense. They also stated that their motivation and job satisfaction had decreased. Three longitudinal studies from Italy examined the relationship between violence and psychosocial factors [23,32,33]. They found that HCW who were exposed to non-physical violence were subject to high job strain, low support, low perceived organizational justice and high psychological stress [23]. On the opposite, HCW with a job strain at baseline had a significant risk of being subject to aggression [33].

Physical injuries were experienced by 58% of those affected, primarily employees in hospitals and disabled care. In comparison to the results from the 2009 study [11], physical impairments have been shown to have significantly increased and thus also the proportion of those affected who required medical attention after an attack. In this way, between 5.0% and 25.0% of those affected (depending on the sector) required medical attention in 2017, while in 2009 this proportion was only 1.9% to 4.1% (depending on the sector).

### 4.5. Stress Perception

For many employees, violence and aggressive acts appear to be part of day-to-day work in nursing and care professions. In our study, one third of employees stated that they felt under great stress as a result of the violence and aggressive attacks they had experienced. This showed that the more common occurrence of verbal abuse was a risk factor for stress perception. A longitudinal study in Italy showed that HCW who had experienced violence in the previous year were at greater risk of work related stress [32]. Another analysis from magnavita examined HWC from 2003–2009. The findings showed that job strain and lack of social support are predictors for the occurrence of non-physical aggression during the ensuing year. The authors came to the conclusion that work related distress and violence is bidirectional [33].

Good preparation by the institution, however, had a positive effect (OR 0.6, 95% CI 0.4–0.8). Employees in sectors with high prevalence rates for violence, such as hospitals (44%) and residential care for people with disabilities (40%) felt the most stress. By contrast, only 27% felt under great stress at institutions for inpatient and outpatient geriatric care. These results differ from those of the 2009 study. Then, stress perception was highest for employees at occupational health facilities for people with disabilities and in outpatient care (36.9% and 37%, respectively). Although employees in outpatient care were less frequently subject to violence and aggression, they were more likely to feel stressed by it. With the increase in the number of incidents and the lack of social support, the level of stress perception has risen [11]. Franz et al. [17] discovered that the highest stress perception was among staff at nursing homes: 35% of staff felt under high stress there, compared with 27% of staff at psychiatric hospitals and 19% of staff at occupational health facilities for people with disabilities.

### 4.6. Management of Violence within the Institutions

In several countries, health and safety legislation has been passed to emphasize the significance of de-escalation training [6,8,34,35]. In the institutions in our survey, various preventive measures were listed, including guidelines for recommended action, follow-up care discussions, case assessments/supervision, de-escalation training and technical emergency systems. Just under half of the organizations had conducted a risk assessment on the topic of violence. However, 55% of employees stated that their institution systematically recorded such events, and 85% reported cases to their superiors. Of the management staff, 66.7% stated that they reported incidents, with just over half of cases also being forwarded to the accident insurer. According to the management staff, reports to the body responsible for the institution were more common (73.6%). These figures therefore show a clear difference as compared with the 2009 study results. Then, incidents were not systematically recorded and only 41% of cases were reported [11]. This means that the reporting behavior has changed significantly. This seems to indicate that the institutions are dealing more openly with the topic of violence. By contrast, Ferri et al. [20] came to the conclusion that 84% of employees did not report incidents. Only serious incidents involving physical violence were reported. Women were less likely to report incidents [20].

In our study, we discovered a positive correlation between preparation by the employer for potential violent experiences and stress perception, as well as evaluating work capacity. 41.8% of respondents had taken part in de-escalation training. This primarily applied to staff in hospitals and disabled care. In terms of outpatient care, only very few employees had taken part in such training. In hospitals, employees on psychiatric wards were more likely to have taken part in de-escalation training. Having said this, there were no significant differences in the frequency of violent events between psychiatric wards and other general wards. There are studies and reviews that deal with the efficacy of training programs in various sectors of the healthcare workplace [28,36,37]. While it is unclear whether de-escalation training can actually reduce violent attacks, it does change the way that attacks are dealt with and strengthens the feeling of safety among employees [37]. A 2014 review found similar results. A total of nine studies were included, although there was only one study with a good study design. All studies observed increased confidence, feeling of security, improved skills in dealing with violence and better knowledge of the risks among employees. The training, however, did not reduce the number of violent incidents. In addition to training and health and safety measures, a zero-tolerance policy is necessary throughout the entire institution in order to decrease the number of violent attacks [36]. By contrast, the randomized intervention study by Arnetz et al. [28] showed a reduction in the number of violent attacks in the intervention group. The intervention comprised creating an action plan for preventing violent attacks in the relevant departments. Six months after the intervention, the incidence rate of attacks in the intervention group was significantly lower than in the control group (incident rate ratio [IRR] 0.48, 95% CI 0.29–0.80). Even after 24 months, the risk of violent attack was lower in the intervention group than in the control group (IRR 0.37, 95% CI 0.17–0.83) [28].

The results underline the importance of a suitable organization strategy and profession-specific training. The basic requirement for preventive measures to combat aggression and violence in the workplace is a corporate culture that treats this topic openly and systematically. Only once this topic is no longer considered taboo can preventive measures really take effect. If, for example, employees have the impression that their superiors and colleagues might see them as incompetent or over-sensitive if they report violent attacks or harassment, there is no basis for effective prevention.

### 4.7. Impact on Work Ability

Employees in healthcare and welfare are subject to enormous psychological stress as a result of verbal and physical violence. Despite this, the test subjects described their work capacity as good to very good. Only very few people considered leaving their profession. Risk factors for poorer evaluation of work capacity were experiencing violent attacks and increasing age. By contrast, good preparation by the institutions for potential violent and aggressive acts had a positive effect on work capacity. Working in disabled care is also a positive factor as compared with employees in geriatric care and in hospitals. In addition to the frequency of violent attacks, however, psychological work pressures such as low staffing, time pressure in terms of care, etc., may also play a role. The fact that violent experiences can influence work capacity was shown in a study looking at patient aggression and the well-being of nurses. More incidences were listed by psychiatric nurses than nurses on internal medicine and surgical wards. Employees in psychiatric care had a worse general health status and lower work capacity than other groups, although they evaluated their psychological well-being as better [5].

### 4.8. Limitations

As a result of the cross-sectional study design, no causal relationships could be analysed. Translating the results to the entire group of employees in healthcare and welfare is only possible to a certain extent because the response rate was only 40.9% and therefore a selection bias cannot be ruled out. The likelihood of a selection bias is highest in hospitals, as this is the most heterogeneous group with sometimes very different departments.

The violent attacks of the previous twelve months were recorded using data from the respondents.

The related information is based on self-reported data disclosed by the participants and could not be verified by an objective documentation of incidents.

This means that a recall bias is possible, which could have led to an overestimation of prevalence. In recent years, violence prevention has come to the fore particularly in the health professions in Germany. This has changed the perception of employees on this topic. Violence und Aggression are no longer considered part of the job. This may have led to overestimation. It is also not possible to check what the individual has experienced as violence, but the recording of the experiences of violence is based on a validated instrument.

In return, however, minor attacks may not have been perceived as being violent at all, which would result in an underestimation. The underlying ILO definition of violence at the workplace reflects the breadth of the topic. We examined the stress perception of HCW due to violent experiences. One third of the HCW in our study were exposed to high stress levels. As our study is a cross-sectional study and longitudinal data are not available, it could not be investigated whether increased workloads led to increased attacks as the Magnavita study [33] has shown or whether the violent attacks have led to increased stress. This must be investigated in a longitudinal study.

## 5. Conclusions

The results prove that, in comparison to the 2009 study, the prevalence of physical and verbal violence has increased and is part of many HCWs daily working lives.

As was the case in 2009, one third of respondents feel under great stress as a result. However, it is apparent that the way this issue is addressed in the various workplaces has changed. Where it was previously stigmatized, the topic is now discussed openly and preventive services such as de-escalation training are offered. The fact that the topic is dealt with openly is also reflected in an increase in the number of reports. Over half of the respondents said that cases would be systematically documented now.

Whether there has actually been an increase in the number of violent and aggressive attacks by patients, clients, and residents cannot be determined. The sensitization to the topic has likely resulted in a change in perception of such attacks. What was once considered commonplace in these professions is now seen as something that does not have to be tolerated. This results in higher reporting figures. It has also become clear that good preparation by the institution for such attacks can reduce the stress perception of those affected. Preventive measures such as de-escalation training or first aid support from colleagues are major factors in dealing with and preventing violent and aggressive attacks.

However, the results also show that horizontal violence is a problem in the nursing and care professions, and that targeted preventive measures are important in the institutions themselves.

## Figures and Tables

**Table 1:** **Table1.** Description of study population (*n* = 1984).

	*n* = 1984	Valid %
Gender		
Female	1568	79.4
Male	406	20.6
Age (in years)		
<29 years	417	21.1
30 to 39 years	384	19.5
40 to 49 years	447	22.6
50 to 59 years	592	30.0
>60 years	134	6.8
Type of facilities		
Outpatient care	217	10.9
Inpatient geriatric care	585	29.5
Hospital	594	29.9
Occupational therapy facility	234	11.8
Residential facility	310	15.6
Other	44	2.2
Area of work		
Nursing	600	30.7
Geriatric care	779	39.9
Occupational therapy facility	251	12.8
Residential facility	310	15.9
Other	14	0.7
Occupation		
Certified nurse		
with managerial role	205	10.3
without managerial role	679	34.2
Peripheral healthcare role	413	20.8
Social professions	367	18.5
Interns, trainees, Federal volunteers *	113	5.7
Therapists	72	3.6
Other	135	6.8
Length of employment		
0–5 years	413	21.2
6–10 years	408	20.9
11–15 years	246	12.6
Longer than 15 years	881	45.2
Preparation by institution		
Not at all	539	28.3
Average	677	35.5
Very good	691	36.2
Psychological aggression from colleagues		
Yes	794	40.8
No	1153	59.2
Psychological aggression from relatives		
Yes	73	3.7
No	1887	96.3
Took part in de-escalation training		
Yes	805	41.8
No	1121	58.2
Migrant background		
Yes	360	18.1
No	1624	81.9

* German government programme promoting volunteering among young adults.

**Table 2:** Frequencies of physical and verbal attacks for each hospital.

	Physical Violence *n*/%	Verbal Violence *n*/%	Response Rate %
Hospital 1	27/59 (45.8)	45/59 (76.3)	41.5
Hospital 2 *	42/48 (87.5%)	46/48 (95.8%)	53.9
Hospital 3 *	73/99 (73.7)	85/99 (85.9)	52.1
Hospital 4	26/55 (47.2)	34/55 (61.8)	50.0
Hospital 5	51/103 (49.5)	69/103 (67.0)	35.2
Hospital 6	15/28 (53.6)	22/28 (78.6)	70.0
Hospital 7 *	37/47 (78.7)	43/47 (91.5)	47.0
Hospital 8 *	45/65 (69.2)	52/65 (80.0)	46.0
Hospital 9 *	63/90 (70.0)	83/90 (92.2)	30.3

* Hospital includes HCW from psychiatry/geriatric psychiatry wards.

**Table 3:** Types of violence * in the previous twelve months by area of work.

Variable	Outpatient Care *n* (%)	Inpatient Geriatric Care *n* (%)	Hospital *n* (%)	Occupational Therapy Facility for People with Disabilities *n* (%)	Residential Facility for People with Disabilities *n* (%)
Insults	114 (91.2)	419 (90.1)	480 (96.0)	169 (88.5)	240 (90.9)
Threats	31 (24.8)	98 (21.1)	276 (55.2)	61 (31.9)	86 (32.6)
Racist comments	10 (8.0)	39 (8.4)	128 (25.6)	10 (5.2)	15 (5.7)
Sexual harassment	20 (16.0)	84 (18.1)	77 (15.4)	22 (11.5)	17 (6.4)
Hitting	29 (32.2)	189 (40.6)	215 (43.0)	62 (32.5)	116 (43.9)
Kicking	18 (14.4)	121 (26.0)	180 (36.0)	43 (22.5)	86 (32.6)
Biting	9 (7.2)	70 (15.1)	84 (16.8)	26 (13.6)	52 (19.7)
Pinching/scratching	46 (36.8)	249 (53.5)	272 (54.4)	66 (34.6)	130 (49.2)
Use of objects	13 (10.4)	75 (16.1)	184 (36.8)	50 (26.2)	94 (35.6)

* Multiple answers permitted.

**Table 4:** Consequences of verbal or physical violence for the study population by area of work.

	Outpatient Care *n* = 125 (%)	Inpatient Geriatric Care *n* = 465 (%)	Hospital *n* = 500 (%)	Occupational Therapy Facility for People with Disabilities *n* = 191 (%)	Residential Facility for People with Disabilities *n* = 264 (%)
Emotions *					
Annoyance, anger	81 (64.8)	218 (46.9)	479 (95.8)	155 (81.2)	193 (73.1)
Fear, self-doubt	34 (27.2)	150 (32.3)	208 (41.6)	70 (36.6)	105 (39.8)
Disappointment	41 (32.8)	133 (28.6)	163 (32.6)	67 (35.1)	80 (30.3)
Helplessness	48 (38.4)	158 (34.0)	193 (38.9)	71 (37.2)	88 (33.3)
Sadness	34 (27.2)	126 (27.1)	97 (19.4)	37 (19.4)	47 (17.8)
Loss of confidence in dealing with patients/clients/residents	29 (23.2)	80 (17.2)	90 (18.0)	41 (21.5)	63 (23.9)
Physical impairment *	*n* = 20 (%)	*n* = 104 (%)	*n* = 175 (%)	*n* = 63 (%)	*n* = 84 (%)
Pain					
Less than 10 min	8 (40.0)	57 (54.8)	86 (49.1)	24 (38.1)	37 (44.0)
More than 10 min	5 (25.0)	25 (24.0)	87 (49.7)	26 (41.3)	40 (47.6)
Injuries					
Not visible	7 (35.0)	31 (29.8)	55 (31.4)	25 (39.7)	30 (35.7)
Visible	10 (50.0)	52 (50.0)	98 (56.0)	30 (47.6)	60 (71.4)
Did not see a doctor	11 (55.0)	42 (40.4)	81 (46.3)	29 (46.0)	47 (56.0)
Saw a doctor	1 (5.0)	10 (9.6)	43 (24.6)	13 (20.6)	21 (25.0)

* Multiple answers permitted.

**Table 5:** Frequency and odds ratios for verbal and physical violence (*n* = 1984).

Variables		Experiences with Verbal Aggression			Experiences with Physical Aggression		
		Yes *n* (%)	OR	95% CI	Yes *n* (%)	OR	95% CI
Gender	Male Female	326 (96.4) 1144 (93.5)	1 **0.5**	**0.2–0.9**	239 (70.7) 848 (69.4)	10.9	0.7–1.2
Age (To what does the asterisk refer?)	<29 years 30–39 years 40–49 years 50–59 years >60 years	319 (94.9) 289 (94.4) 347 (95.6) 428 (93.4) 88 (88.9)	1 1.0 1.4 0.9 0.5	0.5–2.0 0.7–3.0 0.5–1.8 0.2–1.2	241 (72.2) 227 (74.2) 257 (70.0) 308 (67.2) 56 (58.3)	**1** 1.2 0.9 0.8 **0.6**	0.8–1.8 0.6–1.3 0.6–1.2 **0.3–0.9**
Area of work	Outpatient care Hospitals Inpatient geriatric care Occupational therapy facilities for people with disabilities Residential facilities for people with disabilities General residential/occupational therapy facilities	111 (90.2) 479 (96.6) 433 (93.7) 174 (90.6) 251 (95.1) 30 (90.9)	1 1.7 2.1 1.0 1.9 1.2	0.8–3.4 0.9–4.7 0.4–2.6 0.7–5.1 0.3–4.8	63 (51.2) 379 (76.3) 334 (72.6) 119 (62.0) 181 (68.6) 19 (57.6)	1 **2.7** **2.7** **2.4** **2.7** 2.0	**1.9–4.1** **1.8–4.1** **1.4–4.1** **1.6–4.6** 0.9–4.7
Occupation	Peripheral healthcare role Nurses Head nurses Social professions Interns, trainees, Federal volunteers Therapists Other	226 (90.8) 551 (96.6) 163 (94.8) 290 (93.2) 82 (95.3) 51 (96.2) 75 (89.3)	1 1.6 **2.3** 1.4 1.7 5.7 1.0	0.7–3.7 **1.2–4.4** 0.6–3.0 0.5–5.7 0.7–45.5 0.4–2.4	195 (65.7) 436 (76.5) 126 (74.1) 200 (64.3) 61 (70.9) 34 (64.2) 43 (52.4)	**1** **1.5** **1.5** 0.8 1.1 0.8 0.5	**1.0–2.4** **1.1–2.2** 0.5–1.2 0.6–1.9 0.4–1.6 0.3–0.9
